# Demystifying death: a qualitative study using the behavior change wheel framework to explore the palliative care education experiences of doctors, nurses, and community residents

**DOI:** 10.3389/fpubh.2025.1529317

**Published:** 2025-02-07

**Authors:** Guodong Yang, Renxiu Wang, Jun Zhao, Kaiwen Ding, Longhui Xu, Yue Liu, Xiaoxuan Han, Chao Zhang, Cuiping Xu

**Affiliations:** ^1^Hospital Vice President's Office, The First Affiliated Hospital of Shandong First Medical University, Jinan, China; ^2^School of Nursing, Shandong University of Traditional Chinese Medicine, Jinan, China

**Keywords:** palliative care, education, community residents, doctor, nurse

## Abstract

**Objective:**

To explore the palliative care education experiences of doctors, nurses, and community residents.

**Methods:**

A semi-structured interview was conducted with 2 doctors, 8 nurses and 9 community residents in Jinan. Content analysis and behavior change wheel theory were used to analyze the interview content.

**Results:**

Motivation: The road to be taken; Emotional touch of personal experiences; Prepare early; Not now. Capability: Multiple cognition; Need for a topic catalyst; Trust bias; Disconnect between learning and application; Treading on thin ice. Opportunity: Willing but unable; The Need for a larger voice and greater participation.

**Conclusion:**

Community palliative care education requires greater attention. Community residents exhibit diverse perceptions and attitudes toward palliative care, reflecting the influence of personal experiences and sociocultural factors. Innovating the content and format of educational resources and enhancing education for community residents and medical staff will facilitate palliative care.

## 1 Introduction

Globally, with the rising incidence of chronic diseases and the acceleration of population aging, palliative care (PC) is gradually becoming the focus of public health work (1). In China, PC services are mainly undertaken by tertiary hospitals and palliative centers, while nursing homes and community medical services gradually are assuming this role as well (2). In 2022, the Chinese government explicitly proposed to establish a PC service mechanism linking medical institutions, communities and homes, and strengthen public life education within the community context (3).

As PC gradually extends from large medical institutions to grassroots communities, it is necessary to focus on community residents’ awareness of PC. Currently, PC education primarily targets medical staff (4), medical students (5), and terminally ill patients and their families (6), yet education for ordinary community residents remains underdeveloped. Studies in China indicate that public awareness of PC was generally unsatisfactory (7–9), lower than in Western developed countries (10, 11). Many individuals lack a basic understanding of PC, its intended beneficiaries, and access to related resources. Misconceptions, such as equating PC with “giving up treatment” or “premature death” (7) further exacerbate this gap, which not only contradicts the growing demand for PC, but also complicates the delivery of relevant services by medical staff (12).

As the front line of public health services, communities are uniquely positioned to disseminate PC knowledge. However, for these efforts to be effective, it is essential to identify specific misunderstandings and knowledge gaps in the local context. This study aims to examine how community residents view PC education in China.

## 2 Methods

### 2.1 Study design

A semi-structured interview was used to explore the experiences of community residents and medical staff regarding PC education. Interviews were conducted either face-to-face or via telephone. The interview questions (presented in the Supplementary Table S1) were based on the Behavior Change Wheel (BCW) framework (13) and were not modified after 2 pilot tests.

### 2.2 The behavior change wheel

The BCW is a theory-driven intervention development framework, consisting of three layers, with the innermost layer, the COM-B model, serving as the core framework for behavior change. The COM-B model analyzes three components influencing behavior change: Capability (C), Opportunity (O), and Motivation (M). Capability refers to the psychological and physical abilities required by an individual to perform a certain behavior, including the knowledge and skills necessary to perform the target behavior. Opportunity refers to the external factors that promote or hinder an individual’s ability to carry out a certain behavior, such as environmental factors, social influences and resources available to individuals. Motivation refers to the conscious and unconscious processes that stimulate and guide individual behavior, including reflective processes (such as beliefs and intentions) and automatic processes (such as habits and emotional reactions). The COM-B model offers a structured approach to intervention planning by assessing the capability, opportunity, and motivation factors that influence behavior, thereby enabling the development of targeted strategies to achieve desired behavioral changes.

### 2.3 Participants recruitment

Community residents were recruited from five communities in Jinan, Shandong Province, all of which had hosted or participated in PC education activities at least once. These activities were typically conducted in the form of community lectures and health consultations, held in the afternoon and lasting approximately 1 to 3 h per session. The educational content focused on introducing the definition, objectives, value, and importance of palliative care, with particular emphasis on pain management and symptom control for patients with cancer and chronic illnesses. Professional educators delivered thematic presentations using supplementary tools such as slides and handouts, while participants were encouraged to freely ask questions and share their perspectives. Participants were contacted using information collected from past activities, and purposive and maximum variation sampling methods were employed.

Inclusion criteria for community residents: (1) adult; (2) participation in at least one community PC education session; (3) clear speech and logical coherence; (4) voluntary participation.

The medical staff were recruited from the affiliated health centers of the above communities or from the First Affiliated Hospital of Shandong First Medical University, with snowball sampling employed to expand the sample size.

Inclusion criteria for medical staff were as follows: (1) participation in at least one community PC education session; (2) employment at a community health center or tertiary hospital; (3) clear speech and logical coherence; (4) voluntary participation.

### 2.4 Data collection

In this study, two researchers (GDY and RXW) conducted interviews, both of whom had received specialized training in qualitative research. GDY is pursuing a master’s degree with a major in palliative care and has previously served as an interviewer for other research projects. RXW holds a master’s degree and has three years of clinical experience. Before the interviews, the researchers provided a brief overview of the purpose and significance of the study to the participants in order to establish rapport. Neither of the interviewers had any prior connection with the participants.

Offline interviews were conducted in the lounge or conference room, while online interviews were held through Tencent Conference (a teleconference software). Researchers’ backgrounds and experiences could potentially bias data interpretation. To mitigate this effect, two researchers took detailed notes, including the interviewees’ tones and expressions during each interview. All interviews lasted 15–30 min, with an average of 24.3 min. All participants signed an informed consent and agreed to audio recording.

### 2.5 Data analysis

The researchers analyzed the interview transcripts using content analysis (14), identifying key concepts and themes relevant to the research questions. These codes were then categorized and summarized to form the primary research themes. The BCW framework guided the analysis by ensuring that the coding and classification aligned with its key components—motivation, capability, and opportunity.

GDY transcribed the interview recordings on the day of the interviews. The transcripts were then distributed to RXW. GDY and RXW independently read the interview transcripts twice, and marked, classified, and coded the text related to palliative care using NVivo 11.0. All transcripts were rigorously checked for completeness and accuracy.

GDY, RXW, JZ compared the coding results and discussed any discrepancies to ensure consistency and reliability in the final analysis. Any disagreements were resolved through discussion, ensuring a unified and robust interpretation of the data.

### 2.6 Ethics

Ethical approval was obtained for this study (SWYX: NO. 2024-187). Participants had the right to refuse to answer any questions and to freely withdraw from the interview. To ensure anonymity, participants’ names were replaced with letters and numbers in the study, such as R1 for community residents, N1 for nurses, and D1 for doctors, respectively.

## 3 Results

### 3.1 General demographic information of the participants

A total of 9 community residents and 10 medical staff were included in the study (Table 1). The median age of the community residents was 49 years (range: 28–62), with 7 (78%) being female. Five residents (55.6%) had a college degree or higher. Among the 10 medical staff, 2 were doctors and 8 were nurses, including 5 community nurses and 3 clinical nurses. The median age of the medical staff was 37 years (range: 27–48), and 7 (70%) held intermediate or higher professional titles.

**Table 1:** General demographic information of the participants.

Community residents	Age	Gender	Educational background	Medical staff	Workplace	Age	Gender	Educational background	Professional title
R1	52	Female	Middle school	N1	Community	27	Female	Bachelor’s degree	Primary
R2	47	Female	College degree	N2	Community	43	Female	Bachelor’s degree	Intermediate
R3	31	Female	Bachelor’s degree	N3	Tertiary hospital	38	Female	Master’s degree	Intermediate
R4	28	Female	Master’s degree	N4	Community	36	Female	Bachelor’s degree	Intermediate
R5	49	Male	College degree	N5	Tertiary hospital	29	Female	Bachelor’s degree	Primary
R6	60	Female	Primary school	N6	Community	42	Female	Bachelor’s degree	Intermediate
R7	62	Male	High school	N7	Community	32	Female	Bachelor’s degree	Primary
R8	36	Female	College degree	N8	Tertiary hospital	48	Female	Master’s degree	Senior
R9	57	Female	Middle school	D1	Community	35	Female	Master’s degree	Intermediate
				D2	Community	47	Male	Master’s degree	Intermediate

### 3.2 Interview results

A total of 19 rounds of interviews were conducted. The content on community PC education was analyzed, and three themes with 11 sub-themes were identified based on the COM-B model of the BCW framework (Table 2).

**Table 2:** Themes and subthemes resulted from data analysis.

Themes	Subthemes
Motivation	The road to be taken
	Emotional touch of personal experiences
	Prepare early
	Not now
Capability	Multiple cognition
	Need for a topic catalyst
	Trust bias
	Disconnect between learning and application
	Treading on thin ice
Opportunity	Willing but unable
	The need for a larger voice and greater participation

### 3.3 Motivation

#### 3.3.1 The road to be taken

All medical staff believed that PC represented the future of public health and would eventually be extended to the community. The core motivation for community PC education was to align with health policies and equip community residents with the necessary knowledge. As one participant explained, “*Palliative care has been around for a long time, but now it’s really on the agenda*.” (D2). Another participant emphasized, “*There needs to be a place where people can formally talk about death and how to die well*.” (N6).

In addition, the potential value of improving professional skills was also highlighted. By addressing the challenges in community PC education, they looked forward to better performance in the future: “*I learned literature retrieval and SPSS by myself, very practical skills*.” (N5). Another participant demonstrated resilience and determination in tackling novel tasks, saying, “*It’s really, really hard, but if I can get this unprecedented topic done, I’m not afraid to take over another one*.” (D1).

#### 3.3.2 Emotional touch of personal experiences

Personal experiences, often involving witnessing or caring for relatives suffering from incurable illnesses, served as strong emotional motivators to implement or participate in PC education. These experiences were described as distressing and suffocating, yet they reinforced the importance of PC. Some participants recalled, “*I missed my loved one who died of cancer. The last time I saw her, she was yellow all over, her stomach was big, but she was very thin. It was caused by ascites. If she had it (palliative care), she might not have suffered so much.*” (R3). One nurse noted, “*I’ve been there, so people need to really understand palliative care*.” (N1).

In one case, a community resident internalized her pet euthanasia experience, expressing a desire to avoid causing unnecessary pain to herself or others. “*I had my pet euthanized six months ago; it was hit by a car. But maybe one day I’ll go through the same thing, and these are things you must decide for yourself*.” (R8).

Another participant also mentioned incidental exposure to the palliative unit, which provided unexpected insights: “*When I was in the hospital accompanying patients, there was one staying in a single room, usually listened to Peking Opera or did cross-stitch in the room every day. I found it out later (it was a palliative ward), but she looked well*.” (R2).

#### 3.3.3 Prepare early

The lack of preparation for end-of-life decision-making motivated many community residents, particularly the older adult people, to attend lectures for more PC information: “*I’m getting on in years but have not really thought much about death. I just made my last will. I’d just say I’m not afraid, just get off at the station*.” (R7). Specifically, one community resident mentioned the importance of lifelong learning and said, “*Never too old to learn, learn the knowledge of the older adult (Laughter)*.” (R7). He asserted that participation in PC education not only serves as a social interaction, but also as a reflection of *the knowledge the old should learn*, which helped him better understand all aspects of aging life.

Younger community residents focused on the long-term needs and well-being of their older family members, as well as the potential impact of chronic conditions, such as coronary heart disease or chronic kidney disease, on quality of life: “*My older adult family member has some heart issues. They’re healthy for now, but it’s good to learn a bit about it just in case*.” (R4); and “*Life is unpredictable, just be prepared for us and others*.” (R5).

#### 3.3.4 Not now

The medical staff gave several reasons why many community residents resisted PC education, ranging from superstitions to a lack of perceived relevance, with comments like: “*enjoy life when healthy*,” “*just accept it if needed*,” “*feels like a curse*,” and “*I’ll be a joke*.” Despite efforts to provide PC information through educational activities, most community residents remained focused on routine health checks, and many left the lectures midway. “*We held free cancer and palliative care clinics in the square. Not many people came; instead, most of them just wanted to check blood pressure or to get gifts; they were not really interested in what we wanted to do. A lot of people just turned around and left*.” (D2).

### 3.4 Capability

#### 3.4.1 Multiple cognition

Initially, most community residents defined PC as euthanasia or psychological counseling. After community education, terms like “*giving up*” and “*negative*” were replaced by “*caring*” and “*dignity.*” Although they were unable to speak specifically about PC, they viewed it as a good way of relief from sickness and loneliness, or as a healthy concept. As they put it, “*Less painful, less sad. Leave peacefully*.” (R1); and “*It’s just how I want it, I just do not want my spouse and family to be upset*.” (R6).

In general, younger community residents’ views were more conservative. As one explained, “*I’m not sure if I will accept it, but it does have its benefits. I feel that way now, but it might change later*.” (R8). Moreover, they preferred to maintain life and believed that persuading oneself to accept PC was a process of gradual and deepening understanding: “*I still feel a bit sorry, cause accepting this really means leaving this world soon, but if I get treatment, there’s still a small chance to survive for me. Maybe I will not really understand until that day*.” (R3). In addition, one community resident expressed a sense of passive comfort and freedom in restriction: “*I’d rather think about it after the tour of my life, with my family, not lying in a bed*.” (R4), while the older residents were more determined and wished to maintain a final measure of decency: “*It’s acceptable, cannot let the pain afflict me*.” (R9).

Notably, many community residents prioritized health, believing that valuing life were prerequisites for respecting death and receiving PC. Changes in lifestyle habits were often mentioned: “*I gave up milk tea and kebabs, signed up for exercise classes and took my parents for check-ups. The palliative care was advisable, but I could not just see the end*.” (R8).

In addition, several community residents still regarded hospitals and doctors primarily as symbols of life-saving and could not accept the concept of assisting others in achieving a peaceful death: “*That makes sense, but it’s so counterintuitive that I went to the hospital to die (Laughter)*.” (R2). Another opinion was more straightforward, “*Frankly speaking, I think this is a disguised reprieve from death (Shrug)*.” (R4).

#### 3.4.2 Need for a topic catalyst

Almost all participants acknowledged that no formal family discussions about death or PC had occurred. Talking about death with older adult family members was often perceived as unfilial or injustice. As one participant shared, “*Honestly, my mother and I are always best friends. We talk about everything, but this is an exception—some things just end up hurting without us meaning to*.” (R4). Likewise, the older adult feared causing their children unnecessary worry, leaving both sides unprepared to discuss the topic. “*I do not want the kids to overthink it. Let them decide, it’s not up to me (Laughter)*.” (R7).

Participants preferred that others initiate the discussion if they were unclear about how to approach the topic. “*If they want to talk, I’ll talk*.” (R6). Some were triggered by external events, such as the death of a friend: “*One of his (father’s) friends passed away from COPD, and he said at dinner that if he meets the same situation, just let him go*.” (N4).

The strong personalities of some elders also discouraged younger family members from raising such topics. “*I cannot even imagine what it will mean for my strong-willed father if I say this. Feels like the sky is falling if I bring this up*.” (R3).

One nurse compared PC to sex education, highlighting similarities in societal attitudes toward these sensitive topics. Both were subjects acknowledged but rarely openly addressed: “*Everyone wants a good ending, but it just like sex education, people imagine it as a secret that is not public but well-known. It seems that all understanding and cognition will automatically improve with age and practice. In fact, this is unlikely*.” (N4).

Medical staff noted that while many community residents recognized the value of PC, few exchanged ideas. Instead, they often responded with silent nods or waited for others to speak: “*They know it’s meaningful, but they do not want to speak up, just nodded. It should not just be us talking while everyone else sits quietly*.” (N2). The nurses also tried to engage community residents to break the silence by playing an enthusiastic audience. “*I pretended to be a resident to liven up the atmosphere, or else it’s really going to be an awkward silence*.” (N3). Despite collecting suggestions after lectures, medical staff often received little feedback, which made them confused about how to improve the design of subsequent sessions. “*We gathered suggestions for improvement after the lecture but nearly got nothing. Still a long way to go — do we need to do it again*?” (N7).

#### 3.4.3 Trust bias

Although nurses played a critical role in PC education, they lacked the trust of community residents, and were often perceived in traditional roles, such as administering injections and medications. PC education was frequently dismissed as hospital marketing with criticisms like: “*They think nurses only give injections and medicine. Without doctors involved, they do not trust us much, thinking we are just promoting the hospital. They say,* ‘*sounds great, but have you tried it yourself*?’” (N1). Another nurse described bluntly the situation as “*Recognition without respect.*” (N6).

There is a trend that community residents preferred attending lectures when doctors were present. However, due to the term “care,” doctors regarded PC education as the responsibility of nurses and were reluctant to participate: “*They (doctors) do not want to go (give lectures), and we cannot change it*.” (N2).

This stereotype and uneven division of labor lead to dissatisfaction with no reward for hard work, which was reflected in the statement: “*It’s a bit of hard work without deserved appreciation*.” (N5).

#### 3.4.4 Disconnect between learning and application

There was a gap between the teaching materials and the actual needs in community PC education. The cases presented in the textbooks often originated from hospital settings, which differed from the situations in communities and families: “*The current resources are mostly aimed at medical personnel or hospitals, but there’s a need for more content that’s relevant to communities or families. We cannot talk too much about how to treat or use drugs. It’s too dry and too hard for residents to understand and remember*.” (N8). Furthermore, excessive focus on hospital-related content was seen as off-putting to audiences. “*Hospitals are still the main force of palliative care, but who wants to keep an eye on the hospital*.” (D1).

Another challenge in medical staff training was the lack of focus on practical skills due to too much emphasis on theory. As one nurse noted, “*This field is very hands-on, but our training is too much in theory*.” (N3). Furthermore, tools that can concretize PC were needed. “*Like, we can use a model to show how to press ear points. But for palliative care, we do not have any physical examples like that*.” (N4).

Furthermore, medical staff required additional training as they often struggled to address specific inquiries posed by patients, such as the “backfire” they listed below:

“*One person said,* ‘*What if a patient feels bad after getting palliative care, and their life quality goes down, making them die faster*.’” (D1).

“*Last time someone asked me,* “*what is the end-of-life period?*” *I said it was within 6 months, and she asked,* “*if the last palliative care was more than 6 months, did they maybe miss out on treatment, it’s hard to explain to recipient*.” (D2).

“*Like how do we determine whether our family members want to accept it or not? This cannot be a moral kidnapping*.” (N8).

“*She asked if hemodialysis patients still need dialysis in palliative care. I said it have to think about a lot of things because it involved a lot, and then she felt like I was just trying to get by*.” (N3).

#### 3.4.5 Treading on thin ice

Medical staff experienced significant emotional stress when navigating this sensitive topic with caution, which compelled them to be highly attentive to their diction and content, as well as to balance a serious and relaxed teaching style. One nurse observed, “*Most of the people there were old, and some words were hard for them to get, so we had to say things a few times without being too straight, which made it a bit confusing*.” (N8). Any language or prepared content that was too direct or potentially anxiety-inducing was avoided. “*I was afraid they might get upset or think too much, so I left some stuff out. I’ve never been that nervous*.” (N6).

A nurse recalled an incident in which a community resident became emotionally overwhelmed during a meeting due to a seriously ill relative. “*I was really worried about those who had sick family members because someone cried last time. We had to take her to another room and calm her down*.” (N7).

Medical staff emphasized the importance of emotional guidance and gradual teaching. They recommended exploring more inspirational topics, such as the meaning of life and ethical decision-making. Rather than focusing solely on the specific PC details, they suggested: “*You should not just jump into the main topic right away, especially with older folks. It’s not about how much they learn, but more about getting what palliative care is; they need guidance, not memorization of knowledge*.” (D1), an approach which helped community residents understand the value of PC from a broader perspective and facilitated a shift in their views on life and death. One community resident reflected, “*I cannot recall the details; it’s more about the change in mind, like how we think about life and death. I never thought that*.” (R1).

### 3.5 Opportunity

#### 3.5.1 Willing but unable

Older adult individuals with illnesses were the main education target, but due to physical conditions, many of them, along with their caregivers, were unable to attend. “*Those who really need palliative care cannot come, their bodies do not allow them*.” (D2). Additionally, hearing impairments or heavy accents often made it difficult for some to fully understand the content. As reported by a participant, “*Someone cannot hear well, or have accents, it’s hard to talk to each other*.” (D2).

Similarly, young community residents often encountered scheduling conflicts due to work or other obligations, making it difficult to attend lectures consistently. “*I just listened a little, about 30 min, then it ended. I have to Google it myself*.” (R3).

#### 3.5.2 The need for a larger voice and greater participation

Most community residents claimed that they were previously unaware of PC, with two residents indicating that their initial impressions were shaped by short videos on TikTok. Medical staff emphasized the necessity for government efforts to promote PC policies, helping community residents establish a cognitive framework and proper mental preparation: “*The promotion just is not there; lots of people do not know or understand, and some do not even believe it*.” (N8). Another staff member drew a simple comparison: “*People do not really know what palliative care is, unlike they do with diabetes or hypertension. So, the government should help folks get a basic understanding. Then, when we talk about it, it will not seem so surprising*.” (D1). Advocating for broader societal recognition of PC, one community resident stated: “*Just step up publicity so that our families will not think that this is just a personal will, but a social consensus*.” (R5).

Community residents also proposed specific suggestions for educational formats. They emphasized the need for interactive and diverse approaches, authoritative facilitators, and appropriate session frequency: “*This is a controversial topic, but I hope it can have more relaxing elements, so people can be attracted and think about it. But do not be too frequent, it will cause more people’s disgust*.” (R4). Another community resident remarked, “*I do not want to sit in the classroom anymore. It’s easy to get sleepy*.” (R5).

Similarly, universities and tertiary hospitals were identified as significant forces in advancing community PC education efforts. One medical staff noted, “*It’d be great if we could get universities or hospitals involved. They’ve done volunteer work before that people really liked*.” (N2).

PC volunteer teams were also mentioned as a potential resource for enhancing outreach and engagement. As a doctor suggested, “*The Morning Star (a local palliative care volunteer team), they got a lot of praise online. I think it’s good if they can get involved in this kind of work*.” (D1).

Finally, digital media was seen as a key platform for spreading awareness while ensuring personal privacy. “*If palliative care can be spread on TV series or short videos, people might chat more about it in anonymity*.” (R4).

## 4 Discussion

This study investigated the educational experiences in community PC from various perspectives, contributing to an understanding of the slow implementation of PC. PC was inherently regarded as desirable; however, several factors impeded the preparation for PC, including varying degrees of unpreparedness, incapacity, and disbelief regarding death and end-of-life matters, which were influenced by personal experiences, culture, and disparities in educational and medical resources.

### 4.1 Motivation

The implementation of community-based PC education reflects multiple motivations, including the transmission of knowledge, the implementation of policies, and the enhancement of professional skills, and is considered a process that benefits both altruism and self-interest. In the context of an aging population (15) and the rising incidence of cancer in younger people (16), the popularization of PC holds practical significance.

Other studies also capture the impact of personal experiences, revealing a desire for dignity and comfort and eliciting empathy from community nurses (17). Deep reflections on future life help to address anxiety about death and promote positive attitudes towards self-improvement, lifelong learning, and preparing for the future (18). Higher self-efficacy is also associated with more proactive learning behaviors (19).

Consistent with our expectations, reluctance to engage in PC education is widespread. Community residents who refuse to participate in PC education often adhere to a “better safe than sorry” mentality. When people are healthy, PC is a distant or irrelevant issue, which is more common among young people. The absence of death education and cultural taboos directly hinder individuals’ understanding and coping with death, the dying process, and the impact of death (20). The starting point for death education should be to confront the issue of death directly. Existing studies have improved understanding of death through direct visits to funeral homes (21). Cultivating the ability to think about and discuss death could be attempted in parallel with PC education (22), however, in the cultural context of China, how to explore Chinese death cultural resources as a foundation for shaping views on life and death deserves further research.

### 4.2 Capability

Similar to other studies, community residents often associate “giving up” with PC (23), but this perception can be altered through education, repositioning it as an acceptable means of relief. Young community residents believe that a meaningful death is contingent upon a meaningful life, viewing this as a prerequisite for accepting PC. Erikson’s theory of psychosocial development seems to explain the age-related differences in perceptions of PC (24). The views of young community residents on PC may encompass an exploration of the meaning of life and a fear of the unknown, with a greater focus on immediate gratification and current life. In contrast, older residents may be in the “generativity vs. stagnation” or “ego integrity vs. despair” stages. If they perceive their lives as meaningful, they may be more receptive to PC to maintain dignity and comfort at the end of life.

More importantly, due to the context of avoiding death, some cognitive shifts occur at a slow pace, like ending life in a hospital going against common sense. Individuals need not only to address the challenge of death by enhancing the quality of life but also to imbue life with profound meaning through desensitization to death (25).

The research indicates that appropriate education is both effective and necessary, yet cognitive and attitudinal shifts often manifest as silent agreement, a phenomenon also seen among medical personnel. Luo et al. define the ability to discuss death and explore the meaning of life as “death discourse literacy,” emphasizing the development of knowledge, beliefs, and behaviors (26). There is more concern about how to talk about death than about death itself. In family settings, traditional values of filial piety and care for younger generations dominate. The deficiency of communication skills, uncertainty about others’ willingness to engage, and social taboos create a vicious cycle, making medical personnel feel they are imposing education rather than facilitating dialogue. The absence of emotional feedback may delay decision-making or lead to outcomes contrary to the individual’s wishes (27). To address this, creating a more interactive environment and conducting education within the family unit may be key (28). Medical staff can offer a neutral perspective, helping both parties find the right timing and approach for discussion (29).

Despite nurses being recognized as key providers of PC education, our findings reveal that their roles are often misunderstood by community residents, who typically view them as merely administering injections and medications, which may stem from prejudices of low education and limited job responsibilities. This i3nsufficiency of trust, as shown in our study, aligns with Herzberg’s Two-Factor Motivation Theory (30), which posits that an imbalance between effort and reward can reduce work engagement. When nurses are not recognized for their educational roles, they may feel disengaged, reinforcing the importance of clear role delineation in community-based education. The misunderstanding of nurses’ roles, especially in PC education, further underscores the need for a more collaborative approach and clearer role communication within multidisciplinary teams (31).

Choosing PC involves complex issues, such as respecting patient autonomy and balancing benefits and risks, requiring tailored information to meet community residents’ needs. Additionally, there is a lack of theoretical guidance and appropriate teaching materials in current community PC education, highlighting the need for more rigorous interventions (32). Conveying PC information requires consideration of residents’ emotional states and cultural backgrounds (33). Using cultural materials such as videos and books to convey the values of PC highlights the significant advantages of these intuitive resources in emotional communication and educational effectiveness. Therefore, more reflection is devoted to teaching approaches that aim to shift thinking without directly mentioning PC.

### 4.3 Opportunity

Our research indicated that community residents’ participation in PC education was constrained by physical and time limitations. Similarly, Iris et al. highlight that health issues and physical limitations often result in missed educational opportunities (34). These barriers suggest that educational content and activities need to be more flexible and varied to better accommodate the needs of vulnerable groups. Conducting PC needs screenings before education can better identify individuals who require services (35, 36) and assess their understanding of PC (37).

Community residents and medical staff offered several suggestions to make PC education more authentic and engaging, including involving universities, tertiary hospitals, and volunteer teams. This not only helps improve the quality of interventions but also provides valuable feedback for medical and educational institutions to conduct further research. At present, most community education in China mainly consists of lectures or free clinics on weekdays. The traditional teaching mode may lack interactive and personalized considerations, which calls for innovation in content and delivery to meet diverse needs. Using new media to highlight the effectiveness of pilot PC programs can increase societal attention. Cultural institutions like libraries (38), museums (39, 40), and art galleries (41) can use books, images, and art to promote life education through exhibitions and activities, fostering both public engagement and institutional development.

This study found that community residents initially had limited knowledge of PC, with some first encountering the concept through network short videos. Successful promotion requires public awareness. Although PC has made progress, it remains a relatively new concept for most community residents. In addition to social and cultural influences, we infer that the lack of legislation is also a key factor. China currently lacks dedicated legislation for PC, with related documents and regulations scattered across medical and older adult care policies, failing to form a complete and independent system. There remains a significant imbalance between the demand for and access to PC services (42). This may be attributed to the complexity of the PC issue, sharp ethical conflicts, and the difficulty of legislation, as well as a weak societal awareness and insufficient recognition of the necessity for legislation in this area. The government should expedite the formulation and implementation of specialized laws and policies related to PC, ensuring their broad support and effective integration across medical institutions, communities, and all sectors of society.

## 5 Conclusion

Community PC education faces a multitude of challenges, including misconceptions among residents, emotional barriers, inadequate communication skills, and a disconnect between educational content and actual needs. Increased policy promotion, educational activities, and community engagement can enhance public awareness and acceptance, and a cultural shift is required in the discourse surrounding death and end-of-life care.

### 5.1 Limitations

This study has several limitations: (1) The diversity of the medical staff professional backgrounds is limited, perspectives from other disciplines are equally meaningful for this study. (2) This study may be affected by recall bias and age and gender structure. (3) The views of community residents who opposed PC education were relayed by medical staff, which may partly lead to misinterpretation or omission of information.

## Data Availability

The raw data supporting the conclusions of this article will be made available by the authors, without undue reservation.

## References

[1] Hasson F Nicholson E Muldrew D Bamidele O Payne S McIlfatrick S. International palliative care research priorities: a systematic review. BMC Palliat Care. (2020) 19:16. doi: 10.1186/s12904-020-0520-8, PMID: 32013949 PMC6998205

[2] Zhang J Cao Y Su M Cheng J Yao N. Challenges faced by chinese community nurses when providing home-based hospice and palliative care: a descriptive qualitative study. BMC Palliat Care. (2022) 21:14. doi: 10.1186/s12904-022-00905-8, PMID: 35105360 PMC8805229

[3] Liu J Xu S. The historical development, institutional dilemmas, and national policy framework of China's palliative care service system. Soc Const. (2024) 11:3–22.

[4] Artioli G Bedini G Bertocchi E Ghirotto L Cavuto S Costantini M. Palliative care training addressed to hospital healthcare professionals by palliative care specialists: a mixed-method evaluation. BMC Palliat Care. (2019) 18:88. doi: 10.1186/s12904-019-0476-8, PMID: 31655585 PMC6815393

[5] Li S Zhang Z Zhang X. A qualitative study exploring nursing students' perspectives on and attitudes towards hospice care in China. Nurse Educ Today. (2022) 119:105384. doi: 10.1016/j.nedt.2022.105384, PMID: 35750535

[6] Starr LT Washington KT Jabbari J Benson JJ Oliver DP Demiris G. Pain management education for rural hospice family caregivers: a pilot study with embedded implementation evaluation. Am J Hosp Palliat Care. (2024) 41:619–33. doi: 10.1177/10499091231191114, PMID: 37491002 PMC11032627

[7] Lei L Lu Y Gan Q Hu Z Luo Y. Awareness and perceptions of palliative care among the elderly: a qualitative study. J Palliat Care. (2022) 37:204–12. doi: 10.1177/08258597221082393, PMID: 35195464

[8] Yan Y Zhang H Gao W Liu D Endo M Deshpande GA. Current awareness of palliative care in China. Lancet Glob Health. (2020) 8:e333–5. doi: 10.1016/S2214-109X(20)30006-1, PMID: 32087166

[9] Tam KI Che SL Zhu M Leong SM. Knowledge of palliative care and preference of end of life care: a cross-sectional survey of residents in the chinese socio-cultural background of Macao. BMC Palliat Care. (2021) 20:87. doi: 10.1186/s12904-021-00798-z 34158024 PMC8220704

[10] Bergman TD van der Plas A Pasman H Onwuteaka-Philipsen BD. Awareness and actual knowledge of palliative care among older people: a dutch national survey. J Pain Symptom Manag. (2023) 66:193–202.e2. doi: 10.1016/j.jpainsymman.2023.05.005, PMID: 37207787

[11] Noh H Lee HY Lee LH Luo Y. Awareness of hospice care among rural african-americans: findings from social determinants of health framework. Am J Hosp Palliat Care. (2022) 39:822–30. doi: 10.1177/10499091211057847, PMID: 34856830

[12] Ramos K Kaufman BG Winger JG Boggins A Van Houtven CH Porter LS. Knowledge, goals, and misperceptions about palliative care in adults with chronic disease or cancer. Palliat Support Care. (2023) 22:1707–13. doi: 10.1017/S1478951523001141, PMID: 37559194 PMC10858297

[13] Michie S van Stralen MM West R. The behaviour change wheel: a new method for characterising and designing behaviour change interventions. Implement Sci. (2011) 6:42. doi: 10.1186/1748-5908-6-42, PMID: 21513547 PMC3096582

[14] Graneheim UH Lundman B. Qualitative content analysis in nursing research: concepts, procedures and measures to achieve trustworthiness. Nurse Educ Today. (2004) 24:105–12. doi: 10.1016/j.nedt.2003.10.001, PMID: 14769454

[15] Wang H Qin D Fang L Liu H Song P. Addressing healthy aging in China: practices and prospects. Biosci Trends. (2024) 18:212–8. doi: 10.5582/bst.2024.01180, PMID: 38987161

[16] Ugai T Sasamoto N Lee HY Ando M Song M Tamimi RM. Is early-onset cancer an emerging global epidemic? Current evidence and future implications. Nat Rev Clin Oncol. (2022) 19:656–73. doi: 10.1038/s41571-022-00672-8, PMID: 36068272 PMC9509459

[17] Mallon A Hasson F Casson K Slater P McIlfatrick S. Young adults understanding and readiness to engage with palliative care: extending the reach of palliative care through a public health approach: a qualitative study. BMC Palliat Care. (2021) 20:120. doi: 10.1186/s12904-021-00808-0, PMID: 34320961 PMC8320215

[18] Li X He Y. Research on the death psychology among chinese during and after the covid-19 pandemic. Sci Rep. (2024) 14:3005. doi: 10.1038/s41598-024-53673-1, PMID: 38321097 PMC10847439

[19] Remm S Halcomb E Hatcher D Frost SA Peters K. Understanding relationships between general self-efficacy and the healthy ageing of older people: an integrative review. J Clin Nurs. (2023) 32:1587–98. doi: 10.1111/jocn.16104, PMID: 34716612

[20] Chen H Xiao Y Huang X Fan S Wu H Li L. Research on factors influencing chinese parents' support for death education: a cross-sectional survey. Front Public Health. (2024) 12:1285208. doi: 10.3389/fpubh.2024.1285208, PMID: 38481851 PMC10932985

[21] Wang YL Su J. Immersive life and death education teaching—visiting the funeral home. Med Philos. (2022) 43:65–8. doi: 10.12014/j.issn.1002-0772.2022.04.15

[22] Qi XN Yao Q Yu QM Zhao MM Wang Y Zhong ML. Reflections on innovative general education in palliative care in China. Chin Med Ethics. (2024) 37:332–8. doi: 10.12026/j.issn.1001-8565.2024.03.13

[23] Hugar LA Geiss C Chavez MN Gore LR Thirlwell S Reblin M. Exploring knowledge, perspectives, and misperceptions of palliative care: a mixed methods analysis. Urol Oncol. (2023) 41:327.e19–26. doi: 10.1016/j.urolonc.2023.03.016, PMID: 37225636

[24] Orenstein GA Lewis L. Erikson’s Stages of Psychosocial Development. 2022 Nov 7. In: StatPearls [Internet]. Treasure Island (FL): StatPearls Publishing. 32310556

[25] King LA Hicks JA. The science of meaning in life. Annu Rev Psychol. (2021) 72:561–84. doi: 10.1146/annurev-psych-072420-122921, PMID: 32898466

[26] Luo ZP Gao YH. Towards death discourse literacy. Med Philos. (2023) 44:19–23. doi: 10.12014/j.issn.1002-0772.2023.08.04

[27] Fallowfield LJ Jenkins VA Beveridge HA. Truth may hurt but deceit hurts more: communication in palliative care. Palliat Med. (2002) 16:297–303. doi: 10.1191/0269216302pm575oa, PMID: 12132542

[28] Glajchen M Goehring A Johns H Portenoy RK. Family meetings in palliative care: benefits and barriers. Curr Treat Options in Oncol. (2022) 23:658–67. doi: 10.1007/s11864-022-00957-1, PMID: 35316479 PMC8938578

[29] Yeo SL Ng R Peh TY Lwin MO Chong PH Neo P. Public sentiments and the influence of information-seeking preferences on knowledge, attitudes, death conversation, and receptiveness toward palliative care: results from a nationwide survey in Singapore. Palliat Care Soc Pract. (2023) 17:386494103. doi: 10.1177/26323524231196311, PMID: 37719387 PMC10504834

[30] Knoop R. Relieving stress through value-rich work. J Soc Psychol. (1994) 134:829–36. doi: 10.1080/00224545.1994.9923017, PMID: 7869707

[31] Pfaff K Markaki A. Compassionate collaborative care: an integrative review of quality indicators in end-of-life care. BMC Palliat Care. (2017) 16:65. doi: 10.1186/s12904-017-0246-4, PMID: 29191185 PMC5709969

[32] Park S Kim H Jang MK Kim H Raszewski R Doorenbos AZ. Community-based death preparation and education: a scoping review. Death Stud. (2023) 47:221–30. doi: 10.1080/07481187.2022.2045524, PMID: 35275034 PMC9990089

[33] Dudley N Rauch L Adelman T Canham D. Addressing cultural competency and primary palliative care needs in community health nursing education. J Hosp Palliat Nurs. (2022) 24:265–70. doi: 10.1097/NJH.0000000000000882, PMID: 35560145

[34] van der Heide I Wang J Droomers M Spreeuwenberg P Rademakers J Uiters E. The relationship between health, education, and health literacy: results from the dutch adult literacy and life skills survey. J Health Commun. (2013) 18 Suppl 1:172–84. doi: 10.1080/10810730.2013.825668, PMID: 24093354 PMC3814618

[35] Sudhakaran D Shetty RS Mallya SD Bidnurmath AS Pandey AK Singhai P. Screening for palliative care needs in the community using spict. Med J Armed Forces India. (2023) 79:213–9. doi: 10.1016/j.mjafi.2021.08.004, PMID: 36969124 PMC10037045

[36] Wang L Li Y Zhao R Li J Gong X Li H. Development and validation of the home hospice care needs questionnaire for the dying old adult (hhcnq-de) in mainland China. Am J Hosp Palliat Care. (2024) 41:1391–9. doi: 10.1177/10499091231223486, PMID: 38114232

[37] Kozlov E McDarby M Reid MC Carpenter BD. Knowledge of palliative care among community-dwelling adults. Am J Hosp Palliat Care. (2018) 35:647–51. doi: 10.1177/1049909117725725, PMID: 28819986 PMC5842670

[38] Davis-Berman J. Creating a memory book: undergraduate student experiences with end-of-life interviews. Death Stud. (2014) 38:85–90. doi: 10.1080/07481187.2012.725452, PMID: 24517706

[39] Zarrabi AJ Morrison LJ Reville BA Hauser JM DeSandre P Joselow M. Museum-based education: a novel educational approach for hospice and palliative medicine training programs. J Palliat Med. (2020) 23:1510–4. doi: 10.1089/jpm.2019.0476, PMID: 32023145

[40] Lindqvist O Tishelman C. Wa3 room for death—international museum—visitors' preferences regarding the end of their life. BMJ Support Palliat Care. (2015) 5 Suppl 1:A1. doi: 10.1136/bmjspcare-2015-000906.3, PMID: 25960451

[41] Centeno C Robinson C Noguera-Tejedor A Arantzamendi M Echarri F Pereira J. Palliative care and the arts: vehicles to introduce medical students to patient-centred decision-making and the art of caring. BMC Med Educ. (2017) 17:257. doi: 10.1186/s12909-017-1098-6, PMID: 29246258 PMC5732457

[42] Knaul FM Farmer PE Krakauer EL De Lima L Bhadelia A Jiang KX. Alleviating the access abyss in palliative care and pain relief-an imperative of universal health coverage: the lancet commission report. Lancet. (2018) 391:1391–454. doi: 10.1016/S0140-6736(17)32513-8, PMID: 29032993

