# Supplementary material for: Demystifying death: a qualitative study using the behavior change wheel framework to explore the palliative care education experiences of doctors, nurses, and community residents
Source: Front Public Health. 2025 Feb 7;13:1529317. doi: 10.3389/fpubh.2025.1529317 (PMC11842437; doi:10.3389/fpubh.2025.1529317)
Supplement: Supplementary file 1 [file Table_1.docx]

**Interview outline (community residents)**

| **COM-B** | **Question** |
| --- | --- |
| Capability | How well are you informed about palliative care, and has there been any change after attending a community palliative care lecture? |
| Capability | What additional knowledge or skills do you think you need in the area of palliative care? |
| Opportunity | What obstacles have you encountered when participating in palliative care educational activities? |
| Opportunity | What aspects do you think need improvement to enhance the accessibility and practicality of educational opportunities? |
| Motivation | What is your main motivation for participating in palliative care education? Have you ever felt that participating in palliative care education is particularly important for some reason? |
| Motivation | How should the content and format of education be adjusted to better meet your needs and expectations? |
| Others | Do you have anything else to add? |

**Interview outline (medical staff)**

| **COM-B** | **Question** |
| --- | --- |
| Capability | What insights or gains have you got from conducting palliative care education? |
| Capability | What additional knowledge or skills do you think you need in palliative care education in your professional work? |
| Opportunity | What obstacles have you encountered when participating in palliative care educational activities? |
| Opportunity | What aspects do you think need improvement to enhance the accessibility and practicality of educational opportunities? |
| Motivation | What is your main motivation for participating in palliative care education? Have you ever felt that participating in palliative care education is particularly important for some reason? |
| Motivation | How should the content and format of education be adjusted to better meet your needs and expectations? |
| Others | Do you have anything else to add? |
